# A rare complication of systemic lupus erythematosus in a 9-year-old girl: Questions

**DOI:** 10.1007/s00467-019-04411-7

**Published:** 2019-12-10

**Authors:** Aleksandra Gliwińska, Omar Bjanid, Piotr Adamczyk, Justyna Czubilińska-Łada, Anna Dzienniak, Małgorzata Morawiecka-Pietrzak, Dagmara Roszkowska-Bjanid, Aurelia Morawiec-Knysak, Maria Szczepańska

**Affiliations:** 1 Pediatric Nephrology Ward with Dialysis Division for Children, Public Clinical Hospital No. 1 in Zabrze, ul. 3 Maja 13/15, 41-800 Zabrze, Poland; 2 Department of Pediatrics, Faculty of Medical Sciences in Zabrze, Medical University of Silesia in Katowice, ul. 3 Maja 13/15, 41-800 Zabrze, Poland; 3 Department of Pediatrics, Faculty of Medical Sciences in Katowice, Medical University of Silesia in Katowice, ul. Medyków 16, 40-752 Katowice, Poland; 4 Intensive Therapy And Neonatal Pathology Ward, Public Clinical Hospital No. 1 in Zabrze, ul. 3 Maja 13/15, 41-800 Zabrze, Poland; 5 Pediatric Endocrinology Ward, Public Clinical Hospital No. 1 in Zabrze, ul. 3 Maja 13/15, 41-800 Zabrze, Poland

**Keywords:** Systemic lupus erythematosus, Hyperferritinemia, Hemophagocytic syndrome, Macrophage activation syndrome

## Abstract

Serious renal involvement in systemic diseases is common and generally constitutes a pivotal prognostic factor, making those pathology frequently seen in nephrology departments. Authors describe the case of a nine-year-old girl with lupus nephritis. After admission the patient’s state deteriorated over a period of a few days, with an unremitting high-grade fever, significant weakness and drowsiness, generalized erythema, and decrease of the kidney function to eGFR nadir of 56 ml/min/1,73m ^2^. Treatment with pulsed methylprednisolone was started. After the first pulse the general state of the patient improved slightly, although laboratory tests showed an alarming evolution, with the exacerbation of anemia, leukopenia, neutropenia, increase of serum CRP concentration, extremely high D-dimer concentration and increase in activity of lactate dehydrogenase. The concentration of ferritin rose reaching the level of 540 μg/l, triglicerydes level was also high. Intravenous cyclophosphamide pulse therapy was added to the ongoing steroid treatment, and resulted in a radical patient improvement. Authors underline that it seems important to be aware of rare, non-renal, but potentially devastating complications of systemic diseases, like in this clinical case: the secondary hemophagocytic lymphohistiocytosis (HLH). When HLH complicates a rheumatic disease, it is also referred to as macrophage activation syndrome (MAS). Unfortunately treatment of MAS is still based on reports provided by individual centres and gathered own experiences so drawing up unambiguous diagnostic criteria will be valuable in future. The treatment should be individually tailored, and more specific evidence-based recommendations are needed.

A 9-year-old girl with suspected lupus nephritis was referred with a high-grade fever lasting for 9 days, a small-spotted rash on the lower limbs, and left ankle and right wrist swelling. The patient also reported not “feeling well” for a period of approximately 1 month before the onset of these symptoms, without any other specific complaints. The following laboratory abnormalities were noted before referral: leukopenia and anemia, increased erythrocyte sedimentation rate (ESR) and elevated C-reactive protein (CRP) level, increased d-dimer concentration, positive anti-dsDNA antibodies, erythrocyturia and proteinuria (1.0 g/l). The concentrations of serum creatinine, urea, and uric acid were in normal range. Before the definitive diagnosis could be established, she had received ceftriaxone and antihistamines.

The physical examination on admission to the nephrology ward revealed a butterfly-shaped *erythematous* rash on the face, a well-delineated maculopapular rash on the lower limbs and buttocks, along with cervical, submandibular and inguinal lymphadenopathy. In the laboratory work-up, serum urea and creatinine concentrations were increased, with an estimated GFR of 66 ml/min/1.73 m^2^ according to the revised Schwartz formula. Mild proteinuria and hematuria were found in urinalysis. The complete blood count and blood smear were unremarkable apart from a mild anemia with a positive direct Coombs test, suggestive of possibly immune-mediated hemolysis. Concordantly with the suspicion of lupus nephritis, the concentrations of C3 and C4 complement components were significantly decreased (0.24 g/l and 0.05 g/l respectively). The inflammatory markers (CRP and procalcitonin) were within the normal range. Other noticeable laboratory findings were increased d-dimer and triglycerides (TG) concentrations, as well as elevated activity of aspartate aminotransferase (AST). Ferritin concentration was slightly below, whereas fibrinogen concentration was slightly above the respective reference values (Table 1). ANCA profile, anticardiolipin antibodies and anti-glomerular basement membrane (anti-GBM) antibodies were negative. Virologic tests for HBV, HCV, and EBV as well as blood and urine cultures were also negative. Increased renal echogenicity, splenomegaly, and a moderate pericardial effusion were found in ultrasound.

**Table 1:** The patient’s laboratory measurements

Parameter	At admission	4 days after admission	3 weeks after admission	4 weeks after admission (1 day after kidney biopsy)	5 weeks after admission	Normal range
Hemoglobin (mg/dl)	10.1	9.1	12.2	9.5	11.6	12–15
White blood cell count (× 103/μl)	4.2	1.4	10.9	9.7	7.5	4–10.5
Neutrophils (× 103/μl)	1.7	0.77	6.27	9.5	1.46	1.6–6
Platelet count (× 103/μl)	218	193	461	260	622	150–450
Fibrinogen (mg/dl)	414	256	327	551	-	200–400
d-dimer (μg/ml)	2.5	> 20	0.32	3	0.48	0–0.5
Ferritin (μg/l)	14.9	540	39.7	596	72	20–200
AST (IU/l)	49.2	-	17.1	-	14.6	0–40
LDH (IU/l)	-	711	143	301	-	120–300
Creatinine (μmol/l)	82	52	46	62	43	34–65
Triglycerides (mmol/l)	4.11	4.6	2.2	3.5	4.3	< 1.7
CRP (mg/l)	2.41	40	0.5	50	1.15	0–5
PCT (ng/ml)	0.37	0.6	-	17	0.6	0.02–0.5
Proteinuria (g/l)	Trace	1.38	Negative	Negative	Negative	Negative
eGFR (ml/min/1.73 m^2^)	66	104	118	87	126	> 90

*CRP* C-reactive protein, *PCT* procalcitonin, *eGFR* estimated glomerular filtration rate

After admission, the patient’s state deteriorated over a period of a few days, with an unremitting high-grade fever, significant weakness and drowsiness, the spreading of the rash over the trunk, upper limbs and whole face, progressing ultimately into a generalized erythema, and further decrease of the kidney function to eGFR nadir of 56 ml/min/1.73 m^2^.

A treatment with pulsed methylprednisolone (10 mg/kg) was initiated at this point, despite the lack of a kidney biopsy result, because of the severity of the disease and the strongly suggestive clinical picture as well as auto-antibodies profile, with highly positive antibodies against native SS-A (60 kDa) (+++), recombinant Ro-52 (++), dsDNA (+++), nucleosomes (++), and histones (++).

After the first pulse, the general state of the patient improved slightly, although the skin symptoms were still strongly pronounced, and laboratory tests showed an alarming evolution, with the exacerbation of anemia, leukopenia, neutropenia, increase of CRP concentration, extremely high d-dimer concentration, increase in activity of lactate dehydrogenase, and persistent proteinuria (in morning urine spot). The concentration of TG rose, whereas ferritin concentration increased more than 35-fold, reaching the level of 540 μg/l.

Intravenous cyclophosphamide pulse therapy according to the Euro-Lupus regimen [1] was added to the ongoing steroid treatment and resulted in a radical improvement of the patient’s condition. The fever and skin changes subsided; laboratory test results, including renal function parameters and ferritin concentration, normalized. After 4 weeks, when clinical status was stabilized, a kidney biopsy was performed in order to assess the type and intensity of renal involvement. After the procedure a recurrence of high-grade fever was observed with a raise CRP concentration up to 50 mg/l, PCT 17 ng/ml, d-dimer 3 μg/ml, ferritin level 596 μg/l, and TG 3.5 mmol/l. This early relapse necessitated an intensification of the immunosuppressive treatment (high doses of intravenous steroids) with, again, a quick significant improvement. The kidney biopsy revealed diffuse proliferative lupus nephritis, consistent with class IV G (A/C) according to the ISN/RPS classification, and IV-C according to the WHO classification.

Questions:

1. What is the complication that occurred in this case of lupus nephritis?
2. Are further diagnostic procedures necessary to confirm the diagnosis?
3. What is the optimal treatment of the observed abnormalities?

